# Editorial: Omega-3 fatty acids and immunometabolism in health and disease

**DOI:** 10.3389/fnut.2026.1786217

**Published:** 2026-02-16

**Authors:** Everson Araujo Nunes, Ricardo Key Yamazaki, Gleisson Alisson Pereira de Brito

**Affiliations:** 1 Department of Human Health Sciences, University of Guelph, Guelph, ON, Canada; 2 Federal University of the Southern Frontier, Chapecó, Brazil; 3 Institute of Life and Nature Sciences, Federal University of Latin American Integration, Foz do Iguaçu, Brazil

**Keywords:** ALA α-linolenic acid, DHA - 22:6n-3, EPA - 20:5n-3, fish oil, immune activation, lymphocytes, macrophages, metabolism

Immunometabolism has become a central framework for understanding how metabolic pathways intersect with immune function to influence health and disease. This field reflects increasing recognition that nutrients directly influence immune signaling, inflammatory responses, and metabolic regulation. Among dietary factors, omega-3 polyunsaturated fatty acids have attracted sustained interest because of their capacity to modulate lipid metabolism, immune cell function, and inflammatory resolution. Eicosapentaenoic acid, docosahexaenoic acid, and the plant-derived precursor alpha linolenic acid are now understood to influence these processes across tissues and physiological contexts.

**Figure 1:**
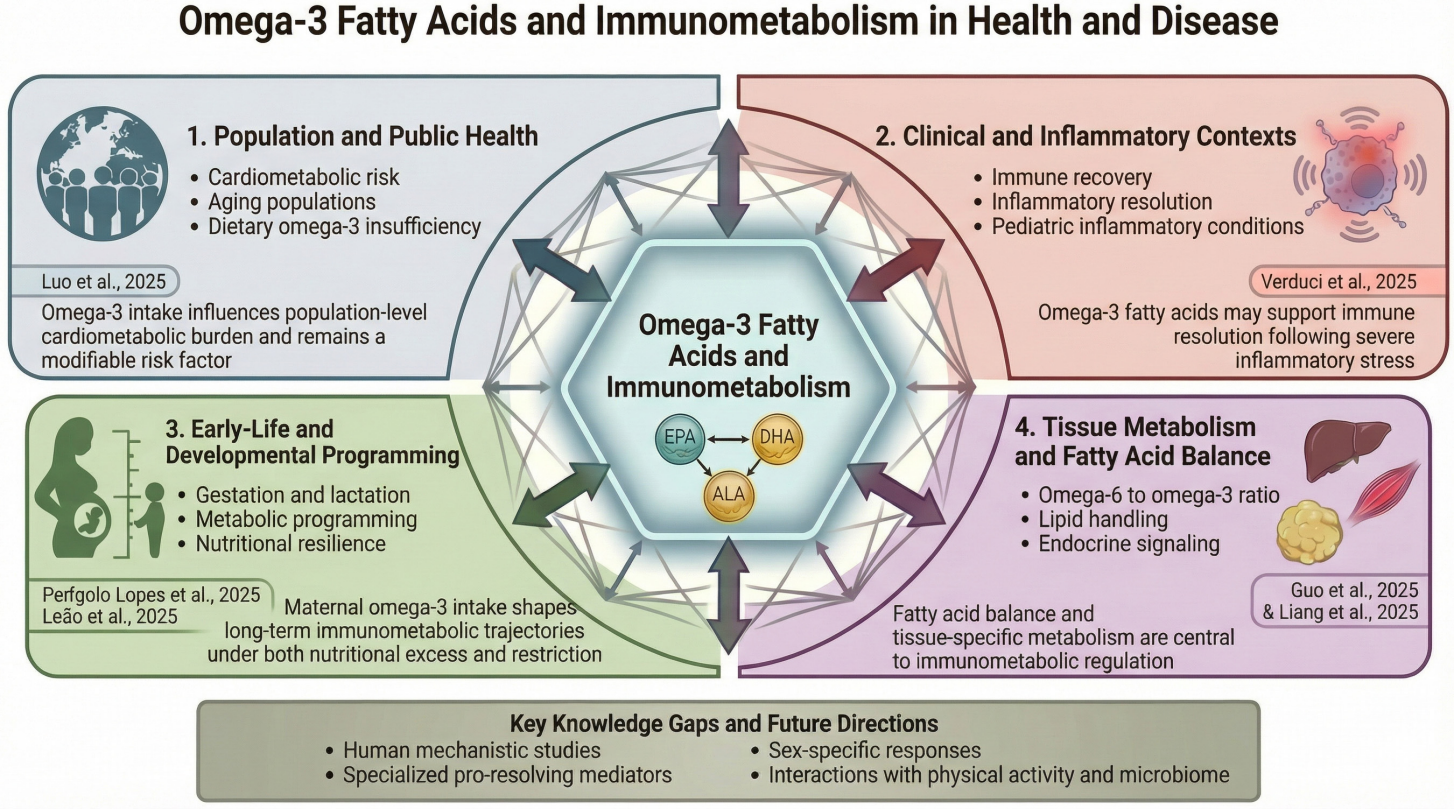
Conceptual framework integrating omega-3 fatty acids and immunometabolism across biological scales.

The Research Topic *Omega-3 Fatty Acids and Immunometabolism in Health and Disease* integrates evidence from population studies, clinical observations, and experimental models to examine how omega-3 fatty acids contribute to immunometabolic regulation. Rather than focusing on isolated outcomes, the included articles collectively advance understanding of shared immunometabolic mechanisms and critical windows during which omega-3 availability appears particularly influential. Across diverse models, studies converge on the idea that omega-3 fatty acids support metabolic flexibility and immune balance in ways that depend heavily on timing, dietary context, and interactions among fatty acids (Figure 1).

At the population level, long-term epidemiological analysis demonstrates that inadequate omega-3 intake continues to contribute meaningfully to cardiometabolic disease burden, even in settings where age-standardized mortality has declined. This relationship is illustrated by recent work quantifying the ischemic heart disease burden attributable to insufficient omega-3 intake across multiple decades and demographic strata (Luo). These findings reinforce the public health relevance of omega-3 consumption and underscore the importance of dietary strategies that account for demographic shifts such as population aging. Importantly, they also provide a necessary backdrop for interpreting mechanistic and interventional studies, linking cellular and tissue-level effects of omega-3 fatty acids to outcomes observed at the population scale.

Clinical relevance is further supported by emerging evidence that omega-3 fatty acids may contribute to immune recovery following severe inflammatory challenges. In pediatric populations recovering from multisystem inflammatory syndrome associated with SARS-CoV-2 infection, supplementation with docosahexaenoic acid has been associated with improvements in circulating omega-3 status and favorable trends in inflammatory markers (Verduci et al.). Although exploratory, these observations suggest that omega-3 fatty acids may contribute to immune resolution during convalescence and highlight the potential for targeted nutritional approaches to complement existing clinical care.

A significant contribution of this Research Topic lies in its emphasis on early life as a sensitive period for immunometabolic programming. Experimental studies demonstrate that maternal omega-3 exposure during gestation and lactation can influence offspring metabolic trajectories, inflammatory signaling, and oxidative balance. In models of post-natal overfeeding, maternal fish oil supplementation attenuated metabolic and inflammatory disturbances in adult offspring (Perígolo Lopes et al.), while complementary work using maternal supplementation with a plant-based omega-3 source showed protection against metabolic impairments induced by post-natal undernutrition (Leão et al.). Considered together, these studies indicate that omega-3 fatty acids contribute to metabolic resilience by shaping developmental trajectories, regardless of whether the early-life nutritional challenge involves excess or restriction.

Beyond absolute omega-3 intake, the studies in this Topic also advance understanding of the importance of dietary fatty acid balance and metabolic context. Experimental manipulation of the omega-6 to omega-3 ratio has demonstrated pronounced effects on lipid handling, endocrine signaling, and tissue-specific metabolic pathways. Evidence from controlled animal nutrition models indicates that reducing the dominance of omega-6 fatty acids improves growth efficiency, lipid profiles, and regulation of metabolic hormones. It modulates gene expression in the liver, intestine, and skeletal muscle (Guo et al.). Complementary mechanistic work further supports the role of omega-3 fatty acids in coordinating metabolic and inflammatory pathways across tissues, reinforcing the concept that omega-3 effects on immunometabolism extend beyond single organs or outcomes (Liang et al.).

Taken together, the contributions in this Research Topic reinforce several key principles relevant to immunometabolism. Omega-3 fatty acids act across tissues and physiological systems to support coordinated metabolic and immune responses. Their effects are shaped not only by dose but also by the timing of exposure, interactions with other dietary fats, and the organism's metabolic state. Early developmental periods and recovery from inflammatory stress appear exceptionally responsive to omega-3 availability, suggesting that nutritional strategies should be context-specific rather than uniform across populations.

Despite these advances, significant gaps remain. The molecular pathways linking omega-3 fatty acids to immune resolution and metabolic adaptation require further clarification, particularly in human studies. The roles of specialized pro-resolving lipid mediators, tissue-specific signaling networks, and interactions with physical activity and the gut microbiome remain incompletely defined. In addition, future research must more effectively address sex-specific responses, interindividual variability, and the long-term sustainability of omega-3-rich dietary patterns in diverse populations. By integrating evidence across epidemiology, clinical research, developmental biology, and experimental nutrition, this Research Topic provides a cohesive view of omega-3 fatty acids as central regulators of immunometabolic health. Rather than presenting isolated findings, the Research Topic emphasizes convergent mechanisms and translational relevance, thereby positioning omega-3 fatty acids within a broader framework of metabolic and immune regulation. Continued interdisciplinary research will be essential to translate these insights into practical strategies for disease prevention and health promotion across the lifespan.

